# A policy analysis exploring hepatitis C risk, prevention, testing, treatment and reinfection within Australia’s prisons

**DOI:** 10.1186/s12954-018-0246-6

**Published:** 2018-08-03

**Authors:** Lise Lafferty, T. Cameron Wild, Jake Rance, Carla Treloar

**Affiliations:** 1 Centre for Social Research in Health, UNSW Sydney, Level 2, Goodsell Building, Sydney, New South Wales 2052 Australia; 2 School of Public Health, University of Alberta, 3-300 Edmonton Clinic Health Academy 11405–87 Ave, Edmonton, Alberta T6G 1C9 Canada

**Keywords:** Hepatitis C, Prisoner health, Treatment as prevention, Policy analysis

## Abstract

**Background:**

Hepatitis C (HCV) is a global public health concern. There is a global prevalence of 15% among the world’s prisoner population, suggesting the need for priority HCV treatment among this population group. New highly efficacious therapies with low side effects, known as directing-acting antivirals, became available under Australia’s universal healthcare scheme on 1 March 2016. This creates an opportune time to trial treatment as prevention as an elimination strategy for HCV in prison settings. This paper examines whether policies in Australian jurisdictions support treatment scale-up to achieve elimination among this priority population.

**Methods:**

A comprehensive search was conducted using Google and other web-based search functions to locate all publicly available policies in each Australian state and territory related to HCV health and HCV-related prison health. Ministers (corrections and health) were contacted from each jurisdiction to identify any additional policies. Inductive and deductive analyses were conducted for each jurisdiction, with documents being assessed against a set of four a priori criteria. Documents included in the analysis were current at 1 September 2017, or 18 months following treatment availability.

**Results:**

A total of 18 documents were located, including both health (*n* = 12) and corrections/prison health (*n* = 6) documents relevant to HCV. Jurisdictions ranged in their commitments for delivering HCV harm reduction strategies and treatment availability within the prison setting.

**Conclusion:**

Few jurisdictions have updated or published HCV-related health or prisoner health policies following availability of directing-acting antivirals. Current policies do not provide effective support for implementing treatment scale-up that could be possible under universal access to HCV treatment among this priority population.

## Background

Hepatitis C (HCV) is an international public health problem with an estimated global prevalence of 1% [1]. HCV is a blood-borne virus and is often transmitted through unsafe injection practices such as sharing of contaminated equipment (in high-income countries) [2, 3]. Due to the criminalisation of drug use, HCV is more prevalent among those incarcerated [4]. Consequently, there is a grossly disproportionate burden of disease related to HCV among those in prison compared with those in the community. There is an estimated 15% global HCV prevalence among the world’s prison population [5], though this rate is higher in Australia at 22% HCV prevalence among prisoner entrants [6].

Previous interferon-based therapy regimes for HCV had high rates of side-effects coupled with low tolerability and low efficacy (54–63%) [7]. The arrival of new direct-acting antivirals (DAAs) has shifted the HCV treatment landscape. DAA treatment regimens are once daily, oral tablets with minimal side-effects and have greater than 95% efficacy [8]. These therapies have been described as ‘groundbreaking’ ([9]:1831), with potential to ‘revolutionise’ HCV treatment ([10]: 1576).

These recent advances in HCV treatment have encouraged a new perspective on reducing prevalence and incidence, i.e. ‘treatment as prevention’ (TasP) [11]. Theoretically, by reducing the number of people who live with the virus, the risk of transmission is subsequently reduced. In settings where access to sterile injecting equipment is not available (such as prisons), TasP has the potential to greatly reduce transmission of HCV among those at risk. Modelling studies suggest that TasP among people who inject drugs in the community could be highly effective in reducing transmission of HCV using DAA therapies when implemented with primary prevention measures such as needle syringe programs and opioid substitution therapy [12, 13]. However, there are no published studies reporting the efficacy of HCV TasP in real-world settings, including prison, although a number of studies are underway in community and prison contexts [14].

On 1 March 2016, DAAs became widely available in Australia with no restrictions on eligibility [15], meaning that, in principle, anyone living with HCV is eligible for treatment irrespective of disease progression as well as current injecting or incarceration status. This treatment-for-all approach counters restrictive treatment practices and legislation common in other countries (see for example, USA [16], Canada [17] and Europe [18]) and provides the basis from which HCV treatments could be delivered at sufficient scale, positioning Australia to assume global leadership in reducing the disease burden of HCV using TasP. Potential benefits are especially possible if TasP is implemented in prisons, where prevalence and incidence are high and concentrated within a defined population [14, 19].

Although TasP has great potential for reducing the population burden of HCV in Australian prisons, the extent to which extant policies support this approach is unknown. To address this gap, the present paper analysed how policies governing TasP in prisons are positioned within and across Australian jurisdictions. This study is necessary to describe policy positions that facilitate or hinder efforts to provide HCV treatment in prisons, at sufficient scale to achieve population health effects [20]. Our focus was thus on high-level policy endorsement of key features of TasP rather than on implementation issues per se. Exploring how different jurisdictions position their policy frameworks on this issue will directly inform the feasibility of any intended TasP scale-up plan [21].

## Methods

### Contextual information on health care governance

In six of eight jurisdictions, prisoner healthcare is delivered by state/territory health departments (or branches of): Australian Capital Territory (ACT), New South Wales (NSW), Northern Territory (NT), Queensland (QLD), South Australia (SA) and Tasmania (TAS) [22]. In two jurisdictions, prisoner healthcare is delivered by state departments of justice/corrective services (Victoria (VIC) and Western Australia (WA)) [22].

### Document retrieval and screening

An iterative search and screening process was used to generate a corpus of policy texts for the present analysis, focusing on all publicly available documents pertaining to Australian policy in three areas of strategic governance, that is, government authorities in each jurisdiction responsible for the provision of (1) public health services, (2) corrective services and (3) health services within correctional facilities.

Google search functions and purposive searches of relevant government department websites (e.g. health, corrective services and corrective health services) were used, according to the following search terms: ‘HCV’ or ‘hepatitis C’ and ‘strategy’ or ‘policy’ or ‘guidelines’ and ‘prison’ or ‘custodial’. Documents were cross-referenced with a previous report regarding prevention of HCV transmission in Australian custodial settings [23] to identify any missing policy documents. To verify the documents obtained via the search process, the peak hepatitis community-based organisation of each state/territory was subsequently contacted. When there were not particular documents from the abovementioned areas of strategic governance, the search was widened to include communicable diseases, mental health, and drug and alcohol policies. Correctional and health ministers from each of the jurisdictions were contacted to confirm the relevant policies had been identified and that no other related policies had been overlooked in our search. The most relevant and comprehensive documents were included from each area of strategic governance.

### Inclusion and exclusion criteria

Policies, strategies and guidelines were assessed as suitable for inclusion if they included focus on treatment of HCV, blood-borne virus and/or prison and prisoner health. Some documents relating to alcohol and other drug use were included given the overlap between drug use and HCV infection and transmission. Documents were required to be current at the time the search was completed (1 April 2017 to 1 September 2017). The relevant department was contacted to confirm the document’s status if information on date of publication was unclear. Policies were excluded if they were less relevant than other health or prisoner health policies within their jurisdiction, guidelines for prescribing treatment, draft versions of policies which have not yet been actioned or endorsed (not yet current), a government act or regulation, no longer current (expired) and program delivery.

Our initial search yielded 34 documents. Retrieved documents were screened for relevance. We defined relevant documents as HCV-related policy texts that were (1) issued by, and representing a state or territorial government, or (2) issued by and representing, a state or territorial delegated health authority, that (3) mandated action regarding management of HCV rates [24].

### Analyses

All included documents were analysed using a two-step process that involved a combination of deductive and inductive approaches. *Deductive analysis* included assessment of each document produced in each jurisdiction using a priori criteria that were applied to assess relative strength of the policies under review with respect to addressing (1) risks of HCV in prison (such as examination of client characteristics and HCV prevalence), (2) strategies for HCV harm reduction and prevention in prison (such as needle syringe programs, opioid substitution therapy and education), (3) HCV testing and treatment modalities in prison and (4) reinfection following treatment in prison. These a priori criteria were developed in consultation with the steering committee of the Surveillance and Treatment of Prisoners with hepatitis C (SToP-C) project. The timeframe of this analysis was relatively close to the introduction of universal access to HCV treatment. Hence, we could not expect policies to have either anticipated this or been updated within the period since the announcement. However, the four criteria are foundational for the implementation of TasP in prison.

Using methods developed for assessing and comparing strength of harm reduction policies across jurisdictions [25], documents were assessed by the authors (LL, JR, CT) as either satisfying (= 1) or not satisfying (= 0) each of the 4 a priori quality criteria. Disagreements were discussed, with a final score achieved through consensus. To facilitate comparisons across jurisdictions, we computed a percentage score across the 4 quality indicators. Specifically, a total policy quality score for each jurisdictions was computed as the sum of all ‘criterion met’ (= 1) scores relative to the total possible score (4 × no. of documents produced in that jurisdiction). The closer a percentage score was to 100%, the more the policy documents produced by the jurisdiction exemplify high-quality policies. Finally, to synthesise our case-level findings and evaluate the overall quality of policies across Australia, we computed percentage scores for each criterion across all 18 documents.

For the *inductive analysis*, relevant text pertaining to HCV (including prevalence, risks, prevention/harm reduction, testing and treatment, and reinfection) and in prison was extracted and analysed, resulting in a set of analytic notes. These notes were synthesised to produce a descriptive summary of the current policies across each of the jurisdictions. Appendix A contains the policy documents included and excluded within this analysis.

## Results

### Documents included in the analysis

Across the eight jurisdictions, a total of 18 documents were included in the final sample. A majority of the documents were from state/territory health departments (*n* = 12), with the remaining (*n* = 6) from prison/prisoner health policies. There were three documents in ACT, two in NSW, none within NT, two in QLD, four in SA, two in TAS, two in VIC and three in WA (Table 1).

**Table 1:** Total number of documents HCV-related health and prisoner health policies by jurisdiction

Case	Total number of current policies included (% of Australian total)
Australian Capital Territory (ACT)	3 (17%)
New South Wales (NSW)	2 (11%)
Northern Territory (NT)	0 (0%)
Queensland (QLD)	2 (11%)
South Australia (SA)	4 (22%)
Tasmania (TAS)	2 (11%)
Victoria (VIC)	2 (11%)
Western Australia (WA)	3 (17%)
Australia	18 (100%)

### Results from deductive analysis

Table 2 presents findings of each jurisdictional report card. The extent to which each of the four criteria were present in jurisdictional documents ranges from 0% (TAS; NT) to 75% (VIC) with an overall score of 47%. The overall presence of criteria was higher for HCV risks, HCV prevention and HCV testing and treatment in prison (61% for each) compared with reinfection (6%).

**Table 2:** Proportion of policy quality indicators endorsed for all documents within cases

Case (number of documents)	Does the set of policy documents mention HCV risks e.g. high prevalence in prison?	Does the set of policy documents mention HCV prevention/harm reduction in prison?	Does the set of policy documents mention HCV testing and treatment in prison?	Does the set of policy documents mention HCV reinfection in prison?	Total score (out of 4 indicators)
ACT (3)	2/3 (67%)	2/3 (67%)	3/3 (100%)	0/3 (0%)	7/12 (58%)
NSW (2)	2/2 (100%)	1/2 (50%)	1/2 (50%)	0/2 (0%)	4/8 (50%)
NT (0)	0/0 (0%)	0/0 (0%)	0/0 (0%)	0/0 (0%)	0/0 (0%)
QLD (2)	1/2 (50%)	1/2 (50%)	0/2 (0%)	0/2 (0%)	2/8 (25%)
SA (4)	3/4 (75%)	3/4 (75%)	3/4 (75%)	0/4 (0%)	9/16 (56%)
TAS (2)	0/2 (0%)	0/2 (0%)	0/2 (0%)	0/2 (0%)	0/8 (0%)
VIC (2)	1/2 (50%)	2/2 (100%)	2/2 (100%)	1/2 (50%)	6/8 (75%)
WA (3)	2/3 (67%)	2/3 (67%)	2/3 (67%)	0/3 (0%)	6/12 (50%)
Australia (18)	11/18 (61%)	11/18 (61%)	11/18 (61%)	1/18 (6%)	34/72 (47%)

### Results from inductive analysis

#### HCV risks in prison

The effective implementation of TasP is predicated on understanding the impact of HCV among prisoner populations, that is, the effectiveness of TasP will be demonstrated by analysis of HCV rates within prison.

Few state and territory health policies across the jurisdictions include discussion of risks associated with HCV transmission in prison. Most discussion in these documents referred to the prevalence of HCV among people in prison and some documents propose prisoners as a priority population for HCV strategies. For example, the *Victorian Hepatitis C Strategy 2016–2020* describes the prevalence in prison as a ‘concentration of chronic’ HCV ([26]:4).

Injecting drug use as a risk factor within prison is mentioned in two policies. The *Queensland Alcohol and Other Drugs Action Plan 2015–2017* notes unsafe injecting use as one of the major risk factors for HCV transmission that disproportionately affects those living in high-risk environments such as prisons [27]. The *South Australian Hepatitis C Implementation Plan 2016–2018* identifies injecting drug use in prisons as a ‘risk behaviour’ [28]. The health policy in TAS does not include risks associated with HCV.

Identified risks of HCV transmission are similar in policies specific to prison/prisoner health with the main inclusion being discussion of prevalence of HCV among prisoners. The *SA Prisoner Blood Borne Virus Prevention Action Plan 2017–2020* is the only document within the national corpus to attribute the high prevalence as reflective of ‘the large number of people entering prison on drug-related offenses and/or with drug dependence issues’ ([29]:9). In VIC, the *Corrections Alcohol and Drug Strategy 2015* identifies that unsafe injecting practices within prison leads to increased health risks, including BBVs [30]. Prison health policies in NSW, NT and WA do not mention HCV risks or prevalence. QLD and TAS do not have HCV-related prisoner health policies.

#### HCV harm reduction in prison

Harm reduction in prison is instrumental to TasP efforts to prevent ongoing transmission and reinfection.

Two states have HCV policies which advocate for access to sterile injecting equipment within the prisons: SA and WA. For example, the *South Australian Hepatitis C Implementation Plan 2016–2018* includes a recommendation to work with the SA Department of Corrective Services ‘to explore barriers for [clean needle programs] in the correctional setting’ ([28]:8). The *WA Hepatitis C Strategy 2015–2018* makes use of an equity argument to outline the need for advocacy ‘for people in custodial settings to have access to the same means of prevention as those in the community, including health hardware such as sterile injecting equipment’ ([31]:8). The WA *Model of Care (2009)* notes that Prison Health Services ‘aim to offer care to prisoners which parallels that which is offered in the broader community’ and includes programs to provide ‘harm minimisation including needle & syringe programs’ ([32]:30). Despite these policy commitments, neither state has implemented needle syringe programs within their respective correctional facilities.

In the HCV health policies of other jurisdictions, harm reduction within the prison context receives little attention. Typically, these strategies call for strengthened harm reduction services but do not make specific calls for these to be in place in prisons. For example, the policies from ACT and TAS mention prevention and harm reduction generically but do not discuss efforts within the prison context. NSW and VIC HCV health strategies do address prison-based harm reduction efforts to some extent. The *NSW Hepatitis C Strategy 2014–2020* identifies the need to ‘strengthen’ existing harm reduction strategies in prisons, including opioid substitution therapy and supporting those who are incarcerated to utilise safer practices ([33]:15). The *Strategy* sets a harm reduction target of reducing sharing of injecting equipment by 25% (although this does not specifically include prisoners) [33]. The *Victorian Hepatitis C Strategy 2016–2020* highlights a number of priority actions to prevent transmission, including increasing awareness and culturally appropriate information dispersal for priority populations (such as those in prison) [26].

Across prison/prisoner health policies, both ACT and VIC mention harm reduction and prevention strategies. The *Strategic Framework for the Management of Blood-Borne Viruses in the Alexander Maconochie Centre 2013–2017* is the only document (inclusive of both health and prison policies) in ACT to include harm reduction/prevention strategies within the prison setting. A priority action of the *Framework* is prevention and education, which includes harm reduction strategies such as provision of bleach and ‘regulated access to sterile injecting equipment’ ([34]:9). However, needle syringe programs are yet to be made available in ACT’s correctional facilities. In VIC, the *Corrections Alcohol and Drug Strategy 2015* includes both education and health promotion as harm reduction strategies as well as screening for BBVs and STIs on reception and transfer between prisons [30]. The *SA Prison Health Service Model of Care* includes a focus on harm minimisation [35], but it is not specific to HCV and does not include HCV among its list of chronic diseases. A recently published policy, the *SA Prisoner Blood Borne Virus Prevention Action Plan 2017–2020*, denotes a section within the policy’s background commenting on the observed effectiveness of prison-based clean needle programs as a preventive measure [29]. However, this recognition is not followed through within the *Action Plan*’s Detailed Strategies and Actions with no commitments for advocacy or other efforts to deliver clean needle programs within the state’s correctional facilities. Prison/prisoner health policies in NSW, TAS and WA do not include mention of harm reduction strategies. QLD does not have any publicly available HCV-related prison/prisoner health policies.

#### HCV testing and treatment

HCV testing and treatment are essential components of any TasP efforts.

Policy documents within five jurisdictions have set clear goals for the identification (such as increased testing) and treatment of those living with HCV in the general population. However, there is a clear absence of focus on HCV in the prisoner population. For example, ACT’s *Statement of Priorities 2016–2020* sets a target of reducing incidence of new infections by 50% and increasing the number of people receiving HCV treatment by 50% each year [36]. Nonetheless, the *Statement* does not indicate inclusion of people in prison within this target. The *NSW Hepatitis C Strategy 2014–2020* aims to increase the number of people accessing treatment by 100% (again, not specifically inclusive of prisoners) [33]. People in custodial settings are listed as a ‘priority population’ group with an action to ‘Prevent’ HCV transmission through building on existing prevention efforts, ‘Manage’ HCV, including increased primary care and best practice management, and ‘Treat’ HCV through improved access to treatment ([33]:5). Although not a specific target, the *Strategy* includes innovative models of care (e.g. nurse-led) in correctional facilities to facilitate increased treatment uptake among those incarcerated. Similarly, the *South Australian Hepatitis C Implementation Plan 2016–2018* sets targets to reduce incidence of new infections by 50% (by 2017) and increase the number of people receiving treatment by 50% each year [28]. The *Implementation Plan* also identifies a priority area to ‘explore, pilot and evaluate new models of care’ such as nurse-led models for priority populations including those in custodial settings ([28]:8). The *Victorian Hepatitis C Strategy 2016–2020* is the only policy within Australia to propose a ‘90-90-90’ target by 2030; 90% reduction in the number of new transmissions, 90% of the proportion of those living with HCV to be diagnosed and 90% of those living with HCV will be cured [26]. The *Strategy* notes that ‘a statewide network of hepatitis clinics has been established throughout the Victorian prison system to ensure prisoners are assessed and treated for hepatitis C’ ([26]:13). HCV-related health policies in WA appear incomplete. Their *Hepatitis C Strategy 2015–2018* outlines goals to improve awareness of treatment options and access to treatment, including within the prison setting, but there are no other policies or strategies proposing how these goals will be achieved. Likewise, WA’s *Hepatitis C Virus Model of Care (2009)* notes that HCV treatment services will be provided in custodial settings [32], but there is no indication as to how these services will be delivered (i.e. telehealth or nurse-led model of care). The health policies of both QLD and TAS do not mention HCV treatment.

The most recent prison policy available across the jurisdictions, the *SA Prisoner Blood Borne Virus Prevention Action Plan 2017–2020*, provides a rationale for providing HCV treatment to prisoners living with HCV. The *Action Plan* contends treatment is likely to ‘achieve a sustained virological response (a cure) which will prevent further transmissions to other prisoners and to members of the community on release. This is known as ‘treatment as prevention’’ ([29]:14). Additionally, the *Action Plan* notes SA Prison Health Service is tasked with ensuring "prisoners are offered testing for relevant communicable diseases" ([29]:4). Unlike other policies within this analysis, the *Action Plan* positions both prisoners and community members as equally benefiting from HCV treatment initiatives (in this instance, TasP). The ACT’s *Strategic Framework for the Management of Blood-Borne Viruses in the Alexander Maconochie Centre 2013–2017* places a restriction on the number of inmates (no more than ten) able to access treatment at any given time [34]. However, it is unclear whether this restriction will be lifted, or revised, in light of the new treatment era. The treatment restriction of the *Framework* is in contrast to the ACT’s *Corrections Management* (*Infectious Diseases) Policy 2014 (No 1)* which notes that all detainees are to receive a health assessment on arrival and to receive treatment and referrals as necessary [37]. The *SA Prison Health Service Model of Care* (2016) exceeds prisoner health policies in every other jurisdiction. The *Model* notes that, with arrival of the new DAAs, ‘there is now an opportunity to significantly reduce rates of hepatitis C amongst prisoners’ ([35]:13). The document outlines healthcare strategies, e.g. telehealth services, to remove barriers to care and increase treatment access for prisoners [35]. VIC’s *Corrections Alcohol and Drug Strategy 2015*’s goal to reduce acquisition and transmission of BBVs and STIs includes strategies for access to management and treatment for HCV and HBV [30]. Additionally, all prisoners testing positive for HCV will be referred to a new statewide hepatitis service, with nurse-led treatment. Prison policies in three states (NSW, TAS, and WA) do not outline targets or goals regarding reductions in transmissions, increases in treatment, or other initiatives relevant to the elimination of HCV. QLD does not have any prison / prisoner health policies.

#### HCV treatment with DAAs

Only three jurisdictions had policies developed after 1 March 2016 regarding, or inclusive of, new HCV treatments: the ACT, VIC and SA. Among these, SA is the only jurisdiction to have a correctional health policy published after the new treatments became available (published August 2016). Due to the low number of policies produced since the provision of DAAs, these results are not categorised into health policies and prison policies.

The first mention of a new HCV treatment regimen on the horizon was in the ACT’s *Strategic Framework for the Management of Blood-Borne Viruses in the Alexander Maconochie Centre*, published in 2013, which notes ‘treatment for hepatitis C is likely to change dramatically over the next few years’ ([34]:11). Despite this early awareness, few policies published in subsequent years incorporated these new treatments into their strategies or goals. The NSW Health’s *Hepatitis C Strategy*, published in 2014, positioned itself as ready to make strides in treatment rollout when DAAs became available, with specific discussion of treatment models in correctional centres to facilitate the increased uptake of treatment [33]. However, no policies could be located within the authority responsible for the provision of prisoner health care in NSW (Justice Health & Forensic Mental Health Network), corroborating commitment to increased delivery of HCV treatment. The *SA Prison Health Service Model of Care*, published in August 2016 (after availability of DAAs), acknowledges the public health benefits of the new therapies in correctional centres, stating that there is ‘now an opportunity to significantly reduce rates of hepatitis C amongst prisoners’ ([35]:13).

Victoria’s policies could be viewed as conflicting in terms of HCV treatment delivery within the correctional setting. While the state’s health body indicates targets for the treatment of those living with HCV (90%) by the year 2030 [26], correctional policies (such as the *Identified Drug User Program*) may present challenges for inmates who inject drugs in prison to come forward for testing, as the admission of being at risk for HCV may compromise the inmate as an ‘identified drug user’. The *Identified Drug User Program* stipulates that inmates who are identified drug users will be invited to join the Drug Free Incentive Program, ‘where they can reduce their time without contact visits in return for extra random urine drug test samples’ ([38]:1).

#### Reinfection

Prevention of HCV reinfection is an important component of TasP efforts in programming support for people during and after treatment to reduce risks and in monitoring these infections to assess these efforts. Policies consistently failed to address HCV reinfection across the jurisdictions. Reinfection is mentioned in the Minister’s Foreword of *Victorian Hepatitis C Strategy 2016–2020*, which outlines that the goal to reduce infections by 90% by 2030 includes both new and reinfections [26]. Reinfection is not mentioned or discussed in any other documents.

## Discussion

This paper described current policies relevant to managing HCV in prisons across each Australian state and territory. Within three possible governance frameworks (health; corrective services; corrective health services), only three jurisdictions (ACT, SA and VIC), produced policies regarding, or inclusive of, new DAA treatments following their inclusion under universal healthcare. Across the four criteria required for TasP implementation (including risks, harm reduction/prevention, testing and treatment, and reinfection), policies in Australian jurisdictions provided only patchy coverage of the key issues suggesting that there is significant opportunity to expand and develop policy frameworks to deliver on the promise of TasP in prisons.

Testing and treatment are critical components of the HCV care cascade in efforts to achieve TasP using DAA therapies. Screening at prison intake is an opportune time to identify people in custody living with HCV [39]. Health providers should closely consider the benefits of an opt-out approach among people entering into custody, thereby likely increasing screening coverage to identify prisoner entrants who may require treatment whilst incarcerated. Clinicians may drive change in the delivery of clinical services in prison to increase the rate of HCV treatment, such as delivery of HCV care and treatment through telehealth and nurse-led models of care [40]. However, other parts of the TasP care cascade, particularly prevention, may not be under the direction or control of prison clinicians [41].

The effectiveness of TasP––the prevention of future HCV infections or reinfection––is predicated on the adequate provision of harm reduction services in prison. Modelling studies have consistently demonstrated that the success of TasP will, in part, depend on the ongoing provision of harm reduction programs (such as needle syringe programs and opioid substitution treatment) [19, 42]. Without access to sterile injecting equipment, such as prison needle syringe programs, or to adequate opioid substitution programs, reinfection remains a risk [4, 19]. In the absence of primary prevention measures, there is greater risk of HCV transmission per injecting incident within the prison (compared with the community) [43]. Prisoners may be reclassified to other areas within a prison, relocated within the correctional system (e.g. transferred to another prison), released to community and, for some, re-incarcerated [44]. This transience, combined with the high prevalence of HCV in the prison setting, requires a focus on effective and accessible prevention strategies [45]. Amid Australia’s policy efforts to eliminate HCV, the absence of focus on reinfection across all jurisdictions (both in prison and the general community) and patchy policy support for primary prevention highlights a critical gap in achieving elimination.

Policies are often a guide, and policies do not always reflect the realities of practice. Changing landscapes (whether healthcare based, such as treatment, or other governance) may alter the practice of policies in place. It follows that organisational and individual level barriers may inhibit HCV treatment access for those incarcerated [20]. Without sufficient consideration of the policy context, too much emphasis may be placed on either the individual level (e.g. deficiencies within inmates’ knowledge or motivation) or the organisational level (e.g. staffing issues) such as in analysis of program effectiveness. While these are important contributors in the delivery and uptake of HCV care, policy analysis provides an understanding of the role of overarching governance frameworks in shaping and guiding organisational processes and subsequent impact. It is reasonable to anticipate that clearly defined policy goals will direct managers’ attention to securing the resources necessary to achieving them. A shortage of nurses in prisons [20], for example, would be incongruent with a policy directive to increase HCV treatment uptake. (Or, conversely, appropriate policy priorities are required if a shortage of correctional nurses is to be adequately addressed.) Innovative models of care, such as telehealth and nurse-led responses, have shown promise in reducing individual and organisational barriers [40]. Nonetheless, such models are only able to be implemented in jurisdictions with supportive policy frameworks. The alignment of policy with strategy is urgent if Australia is to optimise its significant and world-leading investment with the goal of eliminating HCV.

This analysis only includes publicly available policies. While it is likely that other policies exist which were not included in this review, we cannot speculate as to whether these confidential documents might enhance or hinder HCV treatment delivery in the prison setting. It is noted that the NT did not have any publicly available policies regarding HCV within the prison setting at the time our policy search was conducted. An earlier search resulted in one policy from the NT Department of Health, ‘Hepatitis B and C Custodial Care Guidelines 2015’. This document had noted a review date of October 2016. However, at the time of search, the policy was no longer publicly available; our request to the relevant ministers did not produce any additional documents.

The types of documents available across jurisdictions were not uniform. In jurisdictions where there was not a HVC-related health or prisoner health document, we searched for other documents relevant to the research question. It may be that the scores reported on jurisdictional report cards could be influenced by the inclusion of additional documents in some jurisdictions. We felt that their inclusions were warranted to ensure appropriate opportunity for the HCV criteria to be addressed in documents relevant to that jurisdiction. Even with a broader scope of inclusion, it is clear that HCV TasP and reinfection are significant gaps in all jurisdictions.

## Conclusion

Recent biomedical gains, coupled with the Federal government’s decision to subsidise free and universal access to new DAA treatments, have positioned Australia as a world leader in treatment scale-up [46]. Publicly available documents provide a means by which government departments are assessed or held accountable to the statements of intent and purpose in these documents. Analyses such as presented here are key tools to examine existing policy landscapes and to provide direction for future policies. This analysis of the Australian policy environment shows that the current policy infrastructure is inadequate for a general TasP scale-up. Without adequate policies to support treatment scale-up among one of its most ‘at-risk’ populations (i.e. those in prison), Australia is set to fall short of the potential afforded by universal access to HCV treatment. Australia’s position as a world leader risks being compromised if its prisons continue to lack the policy infrastructure required to treat those inmates currently living with HCV.

## Abbreviations

DAA: Direct-acting antivirals
HCV: Hepatitis C virus
TasP: Treatment as prevention

## Australian states and territories

ACT: Australian Capital Territory
NSW: New South Wales
NT: Northern Territory
QLD: Queensland
SA: South Australia
TAS: Tasmania
VIC: Victoria
WA: Western Australia
